# Toxicological Evaluation and Protective Effects of Ethanolic Leaf Extract of *Cassia spectabilis* DC on Liver and Kidney Function of *Plasmodium berghei*-Infected Mice

**DOI:** 10.1155/2022/6770828

**Published:** 2022-02-15

**Authors:** Wiwied Ekasari, Anisah Mahardiani, Nindya T. Putri, Tutik S. Wahyuni, Heny Arwati

**Affiliations:** ^1^Department of Pharmaceutical Sciences, Faculty of Pharmacy, Universitas Airlangga, Surabaya 60115, Indonesia; ^2^Department of Parasitology, Faculty of Medicine, Universitas Airlangga, Surabaya 60132, Indonesia

## Abstract

Currently, the presence of antimalarial drug resistance has become a major obstacle in the treatment of malaria. To overcome the problem, a series of studies are needed to find new antimalarial drugs from plants. Previously, 90% ethanolic extract of *Cassia spectabilis* DC (EECS) leaves have been reported to have antimalarial activity *in vitro* against *Plasmodium falciparum* and *in vivo* against *Plasmodium berghei* ANKA. The research is conducted to find out the toxicity and protective effects of EECS on the liver and kidneys of mice infected with *P. berghei* ANKA. The acute and subacute toxicity tests were carried out on healthy mice that were given EECS at a dose of 150 mg/kg BW. An antimalarial activity test was carried out at doses of 150 and 200 mg/kg BW in *P. berghei*-infected mice. Regarding hepatomegaly, further plasma levels of hepatic enzyme were analyzed, as well as histopathological observation of the liver to determine the effect of the extract on liver. The kidney was observed histopathologically as well. The acute toxicity test of EECS showed that there was no mouse died at the highest dose, indicating safe for the mice. The subacute toxicity based on the histology data showed no significant difference in the liver and kidney of mice between the tested group and the healthy group. The histological and enzymatic effect of EECS in mice infected with *P. berghei* showed the histological and enzymatic effect that improved liver function and the histopathological effect on kidneys with the highest activity at a dose of 200 mg/kg BW compared with the negative control. The results showed the EECS was not toxic in mice and repaired the liver and kidney functions of *P. berghei* ANKA-infected mice, indicating a good candidate for antimalarial drug development.

## 1. Introduction

Until this day, the problem of *Plasmodium* parasitic resistance towards existing antimalarial drugs is still a major problem for the eradication of malaria [1–3]. Therefore, research on the discovery of new sources of antimalarial drugs, one of which is from medicinal plants, continues to be done [4]. *In vivo* and *in vitro* models have long been used in antimalarial testing. In the latter half of the 20th century, *Plasmodium berghei* or *Plasmodium yoelii* was used to infect rodents. Meanwhile, *P. berghei* became the most commonly used species for study at the liver stage, particularly the formation of hypnozoite to investigate malarial recurrence [5–7].

One of the traditional plants in Indonesia, *Cassia spectabilis* DC of the Caesalpiniaceae family, has been proved experimentally *in vitro* against *P. falciparum* and in treating malaria *in vivo* against *P. berghei* [8, 9], indicating that *C. spectabilis* DC plant is very potential to be further developed as a candidate for antimalarial drugs. Previous works reported that an *in vivo* test on 90% ethanolic extract of *C. spectabilis* DC leaf against *P. berghei* ANKA in BALB/c mice showed that an ED_50_ value was 131.5 mg/kg BW [9], and it is categorized as very good antimalarial activity [10]. Furthermore, the extract, fractions, subfractions, and isolated compound of *C. spectabilis* DC have been tested *in vitro* for their antimalarial activities. The active compound of this plant has been successfully identified as a compound that is identical to (–)-7-hydroxycassine, and its *in vitro* antimalarial activity test showed a very low IC_50_ of 0.016 *μ*g/mL [11], which is classified as a very strong antimalarial activity [12].

The research on this plant in relation to overcoming malarial drug resistance has been continued by examining the safety and effects of improving *C. spectabilis* DC leaf extract on the liver and kidney functions in *P. berghei* ANKA-infected mice. Research studies on the effect of medicinal plants that have antimalarial activity on the liver and kidney functions of mice infected with parasites have been widely carried out [13–16], as well as acute and subacute toxicity tests [17–20]. The liver has an important role in regulating physiological processes. This organ is involved in several vital functions such as metabolism, secretion, and storage. In addition, the liver has an important role in the detoxification and excretion of endogenous and exogenous compounds [21–24].

Malarial infection begins when sporozoites are injected by malarial parasite-infected female *Anopheles* mosquito bite. During blood feeding, infected female *Anopheles* injects the sporozoite stage of parasite [25, 26]. After about an hour traveling in human body, sporozoites then enter the liver, attack hepatocytes, and start the asexual cycle of exoerythrocytic schizogony. Inside the liver cells, the parasites multiply asexually, until they reach mature schizont, and finally, a large number of merozoites produce and enter the bloodstream after infecting hepatocyte burst. The infected hepatocytes causing liver damage due to the rupture of infected hepatocytes and merozoites enter the bloodstream and start the erythrocytic cycle in the red blood cells [27, 28]. Damage that occurs in the liver cells can increase the enzymes that work on liver function, especially transaminase enzyme and morphological changes in the appearance of liver [29, 30], such as hepatomegaly. Hepatosplenomegaly is a common feature of malarial infection in humans [31] and mice [32] caused by chronic exposure to malarial parasites. However, no enlargement of kidney in malaria infection.

The dysfunction of the liver can be detected by hepatocellular transamination of plasma glutamic oxaloacetic transaminase (SGOT) and plasma glutamic pyruvic transaminase (SGPT) released in plasma, or by histological examination of the tissue [33]. The most common damage is the activation of apoptotic cell death or hepatocyte necrosis [34–36]. Clinically significant kidney involvement is associated with the infection by *P. falciparum* and *P. malariae*. The infection of *P. falciparum* produces acute manifestations, ranging from asymptomatic, up to urinary disorders, and mild electrolyte disturbances for acute renal failure (ARF) or acute kidney injury (AKI) that require dialysis support [27, 37]. The cases of AKI in the complication of malaria are known to contribute to a high mortality rate, which is about 75% of cases. The histological study suggests the presence of glomerulonephritis, acute tubular necrosis, and interstitial nephritis as the key hemodynamic factor in malaria-associated AKI [38]. Generally, the degree of kidney dysfunction can be detected by the presence of adequate amounts of protein in the urine and an increase in the plasma urea, creatinine, and plasma electrolyte levels [39].

The toxicity of 90% ethanolic extract of *C. spectabilis* DC (EECS) leaves in BALB/c mice, followed by the enzyme examinations, may affect histopathology and liver and kidney functions post-EECS administration.

## 2. Materials and Methods

### 2.1. Plant Material and Preparation of EECS

The *C. spectabilis* DC leaves were purchased and determined in LIPI (Indonesia Research Centre), Botanical Garden, Purwodadi, East Java, Indonesia (B-160/IPH.06/KS.02/III/2019). The specimen was deposited as the herbarium in the Department of Pharmacognosy and Phytochemistry, Faculty of Pharmacy, Universitas Airlangga. The leaves were rinsed thoroughly with tap water to remove extraneous contaminants, dried at 45°C, and ground into powder with a grinder. The extraction was carried out by macerating the powder plant materials (500 g) in a flask containing 2,500 mL of 90% ethanol (at 25–30°C) for 3 × 24 hours. The extraction solvent was separated, filtered through filter paper, and evaporated under reduced pressure by rotary evaporation. The ethanolic extract yield of 1000 g dried weight of *C. spectabilis* DC leaf powder was 10.20% (*w*/*w*) and was used in this experiment.

### 2.2. Animals

Male BALB/c mice aged 6–8 weeks, weight of 25–30 g, were used in the study. All mice were obtained from Farma Veterinary Center, Directorate General of Livestock and Animal Health Ministry of Agriculture, Surabaya, East Java, Indonesia. The animals were housed under standard conditions and fed with a stock diet and water ad libitum. The approval of the study protocol was obtained from the Ethics Committee for Animal Research, Universitas Airlangga, Indonesia, Number 2.KE.181.10.2018.

### 2.3. Acute Toxicity Test

EECS was weighted and resuspended with 0.5% sodium carboxymethyl cellulose (Na CMC) to obtain the desired doses. BALB/c mice were fasted for 24 hours before being fed with the extract. The animals were divided into three groups, each group containing five mice. The doses for each group were 1,250; 2,500; and 5,000 mg/kg BW, respectively. The general behavior of each mouse was observed continuously for one hour after each dose, intermittently every four hours, and thereafter over a period of 24 hours [40–42].

### 2.4. Subacute Toxicity Test

In the subacute toxicity test, mice were grouped into three groups and each group was treated with EECS at once, five times, and ten times of 150 mg/kg BW daily oral dose for 28 days; the normal control group was only given food and drink ad libitum [43]. The animals were observed for 28 days for any sign of toxicity. At the end of observational period, all animals were sacrificed under ether anesthesia and vital organs such as livers and kidneys were removed from all animals for gross and histopathological examinations.

### 2.5. Rodent's Parasite

Chloroquine sensitivity of *Plasmodium berghei* ANKA was obtained from the Institute of Biomolecular Eijkman, Jakarta, Indonesia, and maintained by serial passaging that was used in this study.

### 2.6. Effect of EECS on the Plasma Level of SGOT and SGPT

This experiment was a suppressive test based on Peters [44]. This suppressive test was done prior to the evaluation of the EECS on the plasma level of SGOT and SGPT. The animals were infected with *P. berghei* ANKA and divided into five groups of A, B, C, D, and E. The animals were treated shortly after infection on day 0 (*D*_0_) and continued daily for three days (*D*_1_–*D*_3_). Group A as a negative control received 0.5% Na CMC orally. Groups B and C as the treated groups were given EECS at a single oral dose of 150 and 200 mg/kg BW, respectively. Group D was a positive control given chloroquine at a single oral dose of 100 mg/kg BW. Moreover, group E as a healthy control was not infected with parasites and was only given food and drink ad libitum. On the fourth day (*D*_4_), the blood smears of each mouse were prepared and examined microscopically. Further, plasma was collected prior to the measurement of SGOT and SGPT levels.

### 2.7. Effect of EECS on Histopathology of Liver and Kidney

At the end of the observational period, all animals were sacrificed under ether anesthesia prior to the liver and kidney removal. After weighing the organs, the tissues were then fixed in 10% formaldehyde solution and further processed for hematoxylin-eosin (H&E) staining. The histopathological observation was performed to find out the toxicological effect of EECS on the degenerative and necrotic cells of the tissues. The damages were graded based on Gibson-Corley et al. [45].

### 2.8. Statistical Analysis

The data were represented as mean ± standard deviation (SD). One-way analysis of variance (ANOVA) was used to compare the normally distributed data among the treatments, followed by post hoc multiple comparison test when a different significance was obtained. When the data were not distributed normally, the Kruskal–Wallis and the Mann–Whitney U-test were used to assess the differences among treatments.

## 3. Results

### 3.1. Acute Toxicity Test

No mice died were observed within 24 hours postoral administration of the extract. The behavioral observation of the mice mediated no toxicity sign effects such as paw licking, hair erection, reduction in feeding activity, and increasing respiratory rate [46, 47].

Based on the number of animals' death from the three doses of extract (see Table 1), yielding *r* values were 0, 0, and 0, assuming there was no animal died at the highest dose (5,000 mg/kg BW).

### 3.2. Subacute Toxicity Test

**Figure 1:**
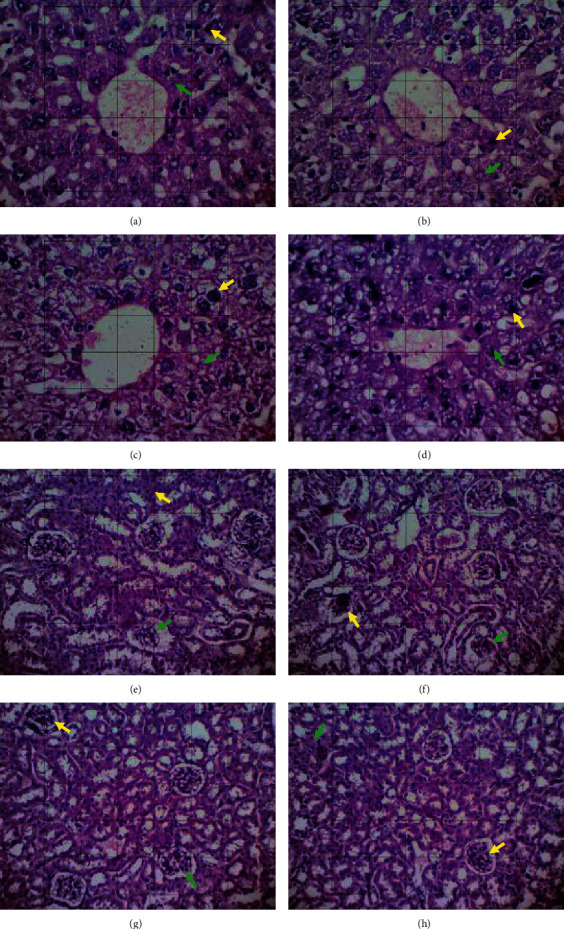
Histological section of the liver (a–d) and kidney (e–h) of mice (section stained with H&E, ×400). Green arrow: degenerative cell, yellow arrow: necrotic cell. (a and e) Single dose. (b and f) Five times of dose. (c and g) Ten times of dose. (d and h) Normal control groups.

The gross examination of the liver showed hepatomegaly, but there was no enlargement of the kidneys. The size and cell morphology of liver and kidney in both the treated and control groups were similar among the groups (see Figure 1). The results of histopathological changes in the liver and kidney tissues are summarized in Table 2. Statistically, there was no significant difference (*p* > 0.05) and histopathological changes were observed in the liver and kidney tissues of the test groups when compared to the control groups.

### 3.3. Effect of EECS on SGOT and SGPT

The effect of EECS on biochemical parameters showed that the EECS intake induced a significant increase in plasma levels of SGOT at the dose of 150 mg/kg BW and was out of the normal range. The normal range of SGOT was 59–247 U/L and SGPT in mouse was 28–132 U/L [48], while the plasma level of SGPT was still in the normal range. At a dose of 200 mg/kg BW, the plasma levels of either SGOT or SGPT were in the normal range. There was a very sharp increase in the plasma levels of SGOT and SGPT in the negative control group, indicating liver damage (see Table 3).

The observations on the morphology of liver and kidneys of mice showed the enlargement of the liver (hepatomegaly) and showed differences in color, size, and texture in the liver. The morphology of kidneys showed no enlargement and was similar either in color, size, or texture among groups. Due to these reasons, the biochemistry examination of the kidney was not performed. Physically, there was an improvement in the liver of mice treated with 150 mg/kg BW of extract when compared to the control, although it was not as good as in mice treated with 200 mg/kg BW of extract. In addition, the appearance of the liver treated with 200 mg/kg BW extract was almost as same as the condition of experimental mouse liver treated with chloroquine and the healthy control group, where brownish-red and smooth texture of surface has appeared.

**Figure 2:**
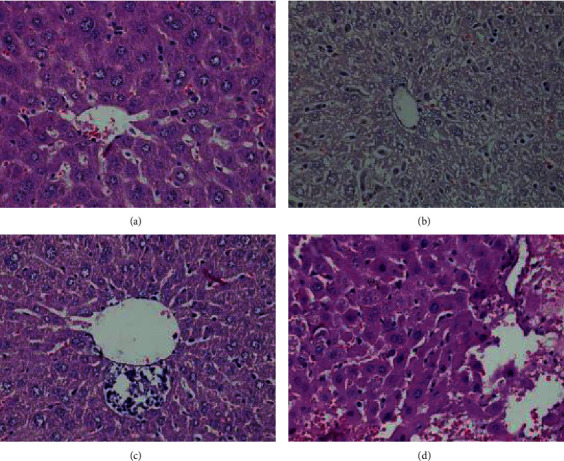
Pathological lesions around the central vein of experimental mouse liver (section stained with H&E, ×400). (a) Normal hepatocyte cells around the central vein. (b) Degenerative nuclear hepatocyte. (c) Inflammatory cell infiltration. (d) Necrotic hepatocyte.

**Figure 3:**
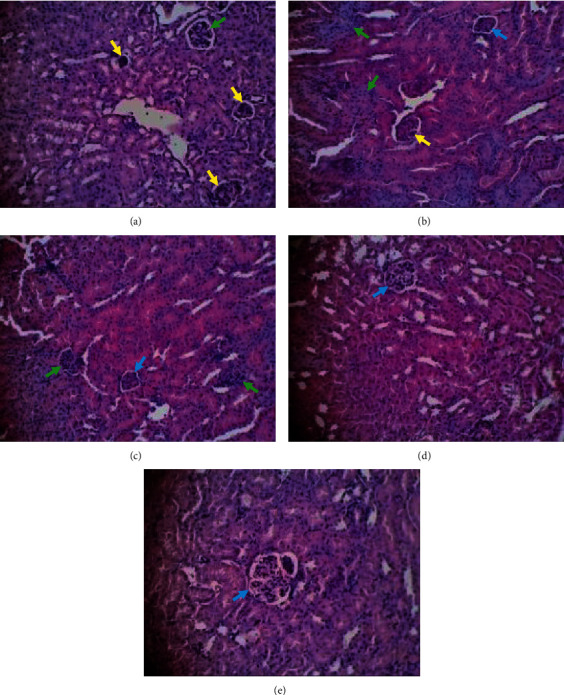
Histopathological appearances of kidney (section stained with H&E, ×400). Green arrow: degenerative cell, yellow arrow: necrotic cell, blue arrow: normal cell. (a) Negative control group. (b) 150 mg/kg BW of extract. (c) 200 mg/kg BW of extract. (d) Chloroquine. (e) Healthy control group.

The results of histopathological changes in the liver and kidney tissues are summarized in Table 4. There were no significant histopathological changes observed in the liver and kidney tissues of the mice when compared to the control. The histopathological changes in liver and kidney are shown in Figures 2 and 3.

## 4. Discussion

Currently, the optimal use of available drugs and the development of a new approach to antimalarial chemotherapy are still needed. With serious consideration for safety issues, researches on phytochemistry, pharmacology, and biochemistry in medicinal plants traditionally used as antimalarial are urgently needed [49]. The plants that show good antimalarial activities that have been recorded until today come from 1,277 plants from 160 plant families [50, 51].

The acute toxicity test of the EECS showed that the EECS is not toxic to the BALB/c mice up to 5,000 mg/kg BW of dose, since there was no mouse died due to this highest dose of EECS. Following this test, the subacute toxicity test showed that there was no abnormality in mouse mobile activities and in water and food consumption.

In addition, the subacute toxicity test was based on the liver and kidney histopathology results (see Figure 1). The data obtained based on microscopic observations were analyzed statistically in the form using the Kruskal–Wallis statistical test to determine the differences in all population groups. Based on the results of the Kruskal–Wallis test, which evaluated liver necrosis damage, it was known that there were no significant differences between the groups. This could be seen from the value of *p* > 0.05. Then, the Mann–Whitney statistical test was also performed to determine the location of differences between the groups. Based on the results of the Mann–Whitney test on the level of liver necrosis damage, it was known that there were no significant differences between the values of *p* > 0.05. Likewise in liver degeneration damage, it could be seen that the four treatment groups showed no significant differences because there were no significant differences (not toxic to the liver).

The suppressive test of the EECS in this current research was performed to find out the effect of EECS on the function of the liver based on the plasma level of SGOT and SGPT. The doses of EECS used in this test were based on the previous suppressive test [9]. Furthermore, the plasma level of SGOT and SGPT in EECS-treated infected mice was maintained in the normal range compared with the controls and based on the reference of normal BALB/c mice [52], especially at the dose of 200 mg/kg BW. The statistical analysis showed no significant difference (*p* < 0.05) in plasma level of SGOT and SGPT when compared with the healthy control group. These results indicated that the EECS was effective in reducing plasma levels of SGOT and SGPT to the normal levels. The excessive hepatic enzymes were released by the injured hepatocytes due to malarial infection [53]. In this study, the necrosis and degraded cells in the liver were repaired by the administration of EECS followed by the reduction in plasma level of SGOT and SGPT. These results indicated a very good liver functional improvement due to the EECS administration.

Apart from blood analysis, the histopathological analysis provides supportive evidence for biochemical and hematological assessment [41]. Histopathological examination is the standard for evaluating treatment for pathological changes in tissues and organs [54]. Histopathological assessment of the effect of EECS on the liver samples treated in this study showed both necrosis and degeneration of hepatocytes and kidney cells (see Table 4). It is likely that these changes may reflect beneficial cellular adaptations of the extract to host tissues.

Based on the results, all doses showed significant differences in kidney lesion scores when compared to the controls (see Table 4). There was a significant difference between chloroquine and the healthy group in necrotic and degenerative cells of kidney. This significant difference shows that chloroquine provides a therapeutic effect on kidney repair in *P. berghei*-infected mice.

In the absence of significant differences between the treated group and chloroquine (see Table 4), it can be concluded that the therapeutic effect is not too different from being used as an alternative to antimalaria, which can reduce kidney damage. Furthermore, a dose of 200 mg/kg BW showed better liver repair activity than a dose of 150 mg/kg BW because there was no significant lesion score in the dose of 150 mg/kg BW compared with the negative control.

The EECS has been proved to be not toxic in mice and was able to repair the liver and kidney functions in mice infected with *P. berghei* ANKA. The ability of EECS in heme detoxification caused the parasite clearance [11] followed by further repairing of tissue in the main organ affected by the malarial infection such as liver and kidney as well as the plasma level of hepatic enzyme.

## 5. Conclusion

The EECS was not toxic in mice and repaired the liver and kidney functions of *P. berghei* ANKA-infected mice similarly to chloroquine, indicating a good candidate for antimalarial drug development.

## Figures and Tables

**Table 1:** Number of animals' deaths in the acute toxicity test of EECS on BALB/c mice in 24 hours.

Doses (mg/kg BW)	Number of deaths (*r*)
1,250	0
2,500	0
5,000	0

**Table 2:** Lesion scores of liver and kidney of BALB/c mice in subacute oral toxicity of EECS.

Organ	Mean score of lesions	Groups				Asymptotic significance (*p* < 0.05)
		EECS 1 × 150 mg/kg	EECS 5 × 150 mg/kg	EECS 10 × 150 mg/kg	Normal control	
Liver	Necrosis	2.34 ± 0.10^a^	2.29 ± 0.30^a^	2.03 ± 0.92^a^	2.37 ± 0.34^a^	0.838
	Degeneration	2.20 ± 0.16^a^	2.43 ± 0.21^a,b^	2.77 ± 0.60^b,c^	2.26 ± 0.25^a^	0.029^*∗*^
	Mean score	2.27 ± 0.13	2.36 ± 0.25	2.40 ± 0.76	2.31 ± 0.29	0.433
Kidney	Necrosis	1.94 ± 0.32^a^	1.09 ± 0.60^b^	1.54 ± 0.63^a,b^	1.60 ± 0.48^a,b^	0.060
	Degeneration	1.14 ± 0.49^a^	1.74 ± 0.19^b^	1.93 ± 0.71^a,b^	1.86 ± 0.46^b,c^	0.065
	Mean score	1.16 ± 0.40	1.41 ± 0.39	1.73 ± 0.67	1.73 ± 0.47	0.062

Data are expressed as mean ± standard deviation (*n* = 7). The superscript (^*∗*^) denotes that the values were significantly different from control (*p* < 0.05; Kruskal–Wallis test for global comparison of organ lesions among groups). The superscripts (a, b, c) indicate that the values in the same row with different superscript letters were significantly different (*p* < 0.05; Mann–Whitney test). EECS: 90% ethanolic leaf extract of *Cassia spectabilis* DC.

**Table 3:** Differences in plasma level of SGOT and SGPT of *P. berghei* ANKA-infected mice treated with EECS compared with the control groups.

Groups	Parasitemia (%)		Suppression (%)	SGOT (U/L)	SGPT (U/L)
	*D*_0_	*D*_4_			
Negative control	1.24 ± 0.69	14.42 ± 7.86	—	658.75 ± 99.52	167.75 ± 32.37
EECS 150 mg/kg	2.13 ± 1.19	8.77 ± 4.42	51.85	384.75 ± 73.98	75.75 ± 35.52
EECS 200 mg/kg	0.98 ± 0.64	9.42 ± 3.27	58.93	216.00 ± 48.80	42.00 ± 4.50
Chloroquine	2.30 ± 1.44	0.14 ± 0.28	100.00	175.50 ± 45.59	41.25 ± 5.60
Healthy control	—	—	—	162.50 ± 59.18	97.25 ± 44.70

Data are expressed as mean ± standard deviation (*n* = 4). No significant difference between the groups (*p* > 0.05; one-way ANOVA followed by Tukey's multiple comparison test). *D*_0_: day before the treatment; *D*_4_: fourth day after treatment; SGOT: plasma glutamic oxaloacetic transaminase; SGPT: plasma glutamic pyruvic transaminase; EECS: 90% ethanolic leaf extract of *Cassia spectabilis* DC.

**Table 4:** Lesion scores of liver and kidney of *P. berghei* ANKA-infected mice treated with EECS.

Organ	Mean scores of lesions	Groups				
		Negative control	EECS 150 mg/kg	EECS 200 mg/kg	Chloroquine	Healthy control
Liver	Necrosis	1.30 ± 0.26^a^	0.75 ± 0.34^a,b^	0.60 ± 0.16^b^	0.65 ± 0.10^b^	0.50 ± 0.12^b^
	Degeneration	2.45 ± 0.38^a^	1.45 ± 0.19^b^	1.10 ± 0.35^b^	1.20 ± 0.28^b,c^	0.65 ± 0.10^c^
	Mean score	1.87 ± 0.32	1.10 ± 0.26	0.85 ± 0.25	0.92 ± 0.19	0.57 ± 0.11
Kidney	Necrosis	1.75 ± 0.50^a^	1.55 ± 0.41^a,b^	1.65 ± 0.19^a^	1.40 ± 0.28^b^	1.20 ± 0.36^c^
	Degeneration	1.30 ± 0.74^a,b^	1.20 ± 0.52^a,b^	1.20 ± 0.16^a,b^	1.10 ± 0.26^a^	1.45 ± 0.68^b^
	Mean score	1.52 ± 0.62	1.37 ± 0.46	1.42 ± 0.17	1.25 ± 0.27	1.32 ± 0.52

Data are expressed as mean ± standard deviation (*n* = 7). No significant difference between the groups (*p* > 0.05; Kruskal–Wallis test). The superscripts (a, b, c) indicate that the values in the same row with different superscript letters were significantly different (*p* < 0.05; Mann–Whitney test). EECS: 90% ethanolic leaf extract of *Cassia spectabilis* DC.

## Data Availability

The data used to support the findings of this study are available from the corresponding author upon request.
